# Coinfection with Dolphin Morbillivirus (DMV) and Gammaherpesvirus in a Spinner Dolphin (*Stenella longirostris*) Stranded in Sri Lanka

**DOI:** 10.3390/v16111662

**Published:** 2024-10-24

**Authors:** Guido Pietroluongo, Claudia Maria Tucciarone, Mattia Cecchinato, Haiyang Si, Luca Spadotto, Işil Aytemiz Danyer, Hewakottege Isuru, Kavindra Wijesundera, Lalith Ekanayake, Cinzia Centelleghe, Sandro Mazzariol

**Affiliations:** 1 Department of Comparative Biomedicine and Food Science (BCA), University of Padua, Viale dell’Università 16, 35020 Legnaro, PD, Italy; guido.pietroluongo@phd.unipd.it (G.P.); mattia.cecchinato@unipd.it (M.C.); luca.spadotto@phd.unipd.it (L.S.); cinzia.centelleghe@unipd.it (C.C.); sandro.mazzariol@unipd.it (S.M.); 2 Department of Animal Medicine, Production and Health (MAPS), University of Padua, Viale dell’Università 16, 35020 Legnaro, PD, Italy; haiyang.si@phd.unipd.it (H.S.); isil.aytemizdanyer@unipd.it (I.A.D.); 3 Department of Wildlife Conservation, 811A, Jayanthipura, Battaramulla 10120, Sri Lanka; 4 Laboratory of Veterinary Pathology, Faculty of Veterinary Medicine and Animal Science, University of Peradeniya, Getambe 20400, Sri Lanka; kavindra77@gmail.com; 5 Bio Conservation Society of Sri Lanka, Kandy 20000, Sri Lanka; lalitheml@yahoo.com

**Keywords:** morbillivirus, herpesvirus, stranding, anthropogenic factors

## Abstract

Following the X-Press Pearl maritime disaster off the coast of Sri Lanka, a stranded spinner dolphin (*Stenella longirostris*) was recovered, and the cause of death was investigated. Post-mortem examinations revealed evidence of by-catch, but a natural coinfection with dolphin morbillivirus (DMV) and gammaherpesvirus was detected by further analyses, marking the first documented case of a dual viral infection in this species within the region. Molecular diagnostics, including PCR and sequencing, were performed on tissue imprints collected on FTA^®^ cards, confirming the presence of DMV in the prescapular lymph node and gammaherpesvirus in the lesions in the oral cavity. The concurrent detection of DMV and gammaherpesvirus raises significant concerns regarding the potential impacts of environmental stressors, such as chemical pollutants from the X-Press Pearl maritime disaster, on exacerbating susceptibility to viral infections in marine mammals. These findings highlight the need for ongoing surveillance of cetacean populations in the Indian Ocean to better understand pathogen circulation and health and conservation implications of anthropogenic activities on the marine ecosystem.

## 1. Introduction

Sri Lanka is characterized by rich marine biodiversity that coexists with intense human activity; therefore, the impact of anthropogenic factors is particularly pronounced. The coastal waters of this island are home to 27 recorded cetacean species [1], which are vital to the marine ecosystem and are increasingly threatened by human-induced pressures. Among these species, the spinner dolphin (*Stenella longirostris*) is the most common odontocete encountered in Sri Lankan waters [2]. Different studies indicate that cetaceans living in this region are exposed to a dramatic increase in human activity; waters surrounding this island are among the ecosystems subjected to the highest predicted cumulative anthropogenic impact globally [3], driven by pollution, fishing, and climate change. For instance, shipping traffic, including whale-watching activities, results in heightened risks of ship strikes and habitat degradation for cetaceans [1], particularly in areas where these animals congregate for feeding and breeding. Among others, entanglement represents one of the most significant anthropogenic threats, with an estimate of 300,000 entangled cetaceans annually worldwide [4], together with marine litter and plastic pollution, affecting marine as well as human welfare [5,6]. The cumulative effects of these anthropogenic stressors have been shown to compromise cetacean health and population viability, also enhancing their susceptibility to various pathogens [7].

Among others, *Cetacean morbillivirus* (CeMV) is a single-stranded RNA-virus species belonging to the genus *Morbillivirus*, subfamily *Orthoparamyxovirinae*, family *Paramyxoviridae*, and order *Mononegavirales*, and it includes different groups of strains: dolphin morbillivirus (DMV), porpoise morbillivirus (PMV), and pilot whale morbillivirus (PWMV) [8]. Like other morbillivirus species [9], CeMV can cause respiratory disease in the acute form, immunosuppression with increased susceptibility to secondary infections in later stages, and neurological diseases, due to its replication in the central nervous system [8]. Clinical disease affects diving and orientation capabilities, becoming an important contributing factor to strandings [10,11,12].

Neurological disorders have also been associated with herpesvirus infections [13,14,15,16,17,18,19], and coinfections with morbillivirus are a common finding due to immunosuppression [20,21,22]; thus, the actual role of herpesvirus has not been defined yet [15,18,19,20]. Herpesvirus in cetaceans can sustain genital [23] and cutaneous infections [24,25], alongside favoring opportunistic infections and likely establishing latency. The interplay between viral agents can exacerbate disease severity, complicating clinical outcomes and management strategies for affected populations.

In this context, Sri Lanka’s biggest maritime disaster [26] occurred on 20 May 2021, compromising an already delicate situation for marine mammal conservation. On that date, the Singapore-flagged container ship X-Press Pearl caught fire off the coast of Sri Lanka, which continued until it sank later on 17 June. During this period, the container ship released dangerous goods into the marine environment, such as chemical substances, fire residues, and plastic pellets (nurdles), impacting marine life and the economic activities in the area [27,28]. Since a consequent increase in marine life mortalities was expected, the Marine Environment Protection Authority (MEPA, Colombo, Sri Lanka) asked for support in investigations on stranded animals. The present study focuses on the diagnostic results for one spinner dolphin (*S. longirostris*), among the examined animals after the incident, that presented a coinfection with DMV and gammaherpesvirus.

## 2. Materials and Methods

### 2.1. Post-Mortem Procedures and Tissue Sampling

During the investigations, cetaceans and sea turtles found stranded in the 6 months following the fire and sinking of the X-Press Pearl container ship [29] were examined. Carcasses were stored at −20 °C until necropsy and defrosted at room temperature for two days before the complete post-mortem examination. Necropsies were performed by trained pathologists from the Department of Comparative Biomedicine and Food Science (BCA) of the University of Padova (Legnaro, Italy) at the Faculty of Veterinary Medicine & Animal Science of the Peradeniya University facilities (FVMAS-UoP, Peradeniya, Sri Lanka) in December 2021, following the standard protocol described in the European “Best Practice on Cetacean Post-Mortem Investigation and Tissue Sampling” document [30]. 

Among the carcasses, a 150 cm long juvenile male *S. longirostris* stranded in Negambo (N 7°7′58.8″, E 79°49′58.799″) on 13 October 2021 was routinely necropsied, considering its decomposition condition category (DCC). The main tissues, including skin, blubber, muscle, liver, lungs, stomachs, intestine, spleen, heart, brain, tonsils, and prescapular and pulmonary lymph nodes, were sampled and preserved in 10% neutral buffered formalin for histopathological examination. 

For microbiological examination, the aliquots of various organs (brain, thyroid gland, thymus, prescapular lymph node, lungs, spleen, liver, kidneys) and lesions (i.e., an ulcer and proliferative lesion observed on the tongue surface) were imprinted as previously described by Si et al. (2023) [31] on QIAcard^®^ FTA^®^ cards (Qiagen, Hilden, Germany) to preserve nucleic acids during long-distance shipment.

FTA^®^ cards and tissue samples were shipped at room temperature to the BCA Department according to the CITES import procedures (CITES permit IT020); then, FTA^®^ cards were transferred to the Infectious Disease Laboratory of the Department of Animal Medicine, Production and Health (MAPS, University of Padova, Legnaro, Italy) for biomolecular analyses.

### 2.2. Histological and Immunohistochemical (IHC) Analyses

All tissues fixed in 10% neutral buffered formalin were dehydrated and embedded in paraffin wax at the facilities of FVMAS-UoP and subsequently shipped to Italy. Sections of formalin-fixed, paraffin-embedded (FFPE) tissues of 3–5 μm were obtained, mounted on glass slides, and routinely stained using hematoxylin and eosin. Furthermore, to remove artifacts related to formalin pigments, all sections were stained using Picric acid [32]. Additionally, skin, blubber, and lung sections were stained using Chromic acid [33] for the identification of fat emboli in the lumen of small vessels, often associated with traumatic findings. Finally, Danscher mercury staining protocol was used on lung and liver sections to detect mercury deposition in the parenchyma [34].

Immunohistochemistry (IHC) for the detection of CeMV was performed on FFPE lung sections according to standard procedures [35]. Briefly, antigen retrieval was performed in citrate buffer, followed by protein blocking in 5% BSA in PBS for 30 min. As a primary antibody, a commercially available mouse monoclonal antibody against Canine Distemper Virus nucleoprotein antigen (Veterinary Medical Research and Development, Pullman, WA, USA, clone NP0505) was used at a dilution of 1:100 in PBS and incubated for 1 h at room temperature. The secondary antibody, a horseradish peroxidase (HRP) conjugated polymer that binds mouse and rabbit primary antibodies (EnVision FLEX/HRP; Dako Omnis, Santa Clara, CA, USA, code GV800), was then incubated for 1 h at room temperature, followed by the application of 3,3′-diaminobenzidine as a chromogen (EnVision FLEX DAB+Substrate Chromogen System; Dako Omnis, code GV825). Sections were counterstained with Mayer’s hematoxylin.

### 2.3. Biomolecular Analyses

Two portions of FTA^®^ cards were cut and preprocessed as previously described by Si et al. (2023) [31]. Then, nucleic acid extraction was performed with the RNeasy^®^ Plus Mini Kit (Qiagen, Hilden, Germany) and the DNeasy Blood & Tissue Kit (Qiagen, Hilden, Germany) following the manufacturer’s instructions. Extracted nucleic acids were stored at −80 °C until further processing.

RNA extracts were screened for DMV using a nested RT-PCR assay published by Centelleghe et al. (2016) [36], and for cetacean coronavirus using a previously validated RT-PCR assay by Legnardi et al. (2024) [37].

DNA extracts were screened for herpesvirus using a nested PCR assay published by VanDevanter et al. (1996) [38]. RT-PCR assays were performed using the SuperScript III One-Step RT-PCR System with Platinum Taq DNA Polymerase kit (Invitrogen™, Waltham, MA, USA); PCR assays were performed using the Platinum™ II Taq Hot-Start DNA Polymerase kit (Invitrogen™, Waltham, MA, USA) on an Applied Biosystems 2720 Thermal Cycler (Applied Biosystems, Waltham, MA, USA). Amplicon specificity was analyzed by agarose gel electrophoresis, and positive samples were Sanger-sequenced with the same primer pair used for amplification.

Chromatogram quality was evaluated with FinchTV Version 1.4.0 software (Geospiza Inc., Seattle, WA, USA), and consensus sequences were assembled using ChromasPro 2.1.8 software (Technelysium Pty Ltd., Helensvale, QLD, Australia). Sequences were preliminary evaluated by BLAST search (https://blast.ncbi.nlm.nih.gov/Blast.cgi (accessed on 8 January 2024)), then merged to databases of available reference sequences downloaded from GenBank. Databases were aligned and phylogenetic analyses were performed using MEGA X software [39]. Sequences were phylogenetically analyzed by reconstructing a Maximum Likelihood phylogenetic tree using MEGA X software, and the substitution model was selected based on the lowest Bayesian information criterion (BIC). Branch support was calculated by performing 1000 bootstrap replicates, and bootstrap values ≥70% were considered reliable.

## 3. Results

### 3.1. Pathological Findings

**Figure 1:**
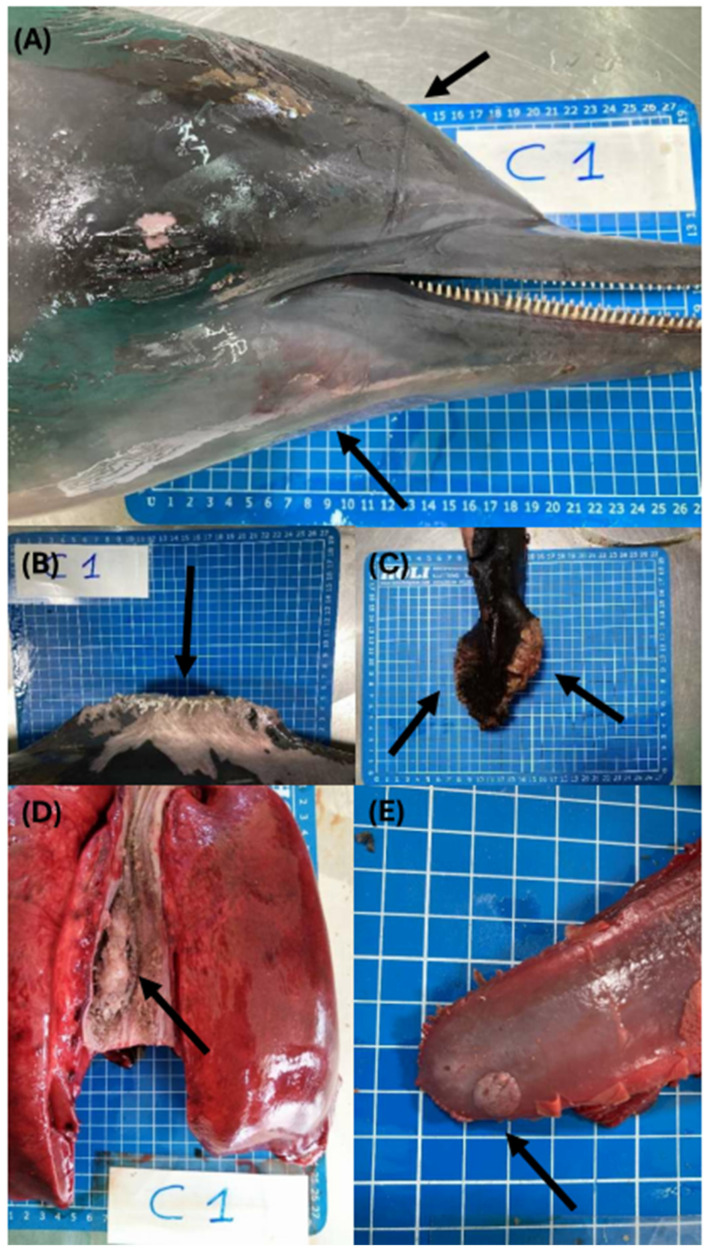
Macroscopic lesions on the carcass: (**A**) linear marks (arrows) from a net impression on the right side of the rostrum; (**B**) amputation of the dorsal fin (arrow); (**C**) amputation of the lobes of the caudal fin (arrows); (**D**) evidence of recent feeding in the esophagus (arrow); (**E**) round, focal, well-defined, and proliferative plaque on the mucosa of the tongue (arrow).

At the time of post-mortem investigation, the carcass was considered fresh (DCC 2), and the animal showed a suboptimal nutritional condition category (NCC 2). During the external examination, evidence of recent interaction with fishing activities (by-catch) was observed (Figure 1A–C); notably, the linear marks of a net impression were evident on the rostrum on the right side of the head (Figure 1A). Additionally, the clear amputation of the dorsal fin and caudal lobes was observed (Figure 1B,C) despite the action of scavengers changing the sharp edges of the traumatic injuries. Finally, evidence of recent feeding was found in the esophagus (Figure 1D) and gastric chambers. Besides the evidence of human interaction, during the observation of the oral cavity, a small, round, focal, well-defined, and raised plaque (Figure 1E), less than one cm in size, was assessed on the mucosa of the tongue, along with two smaller ulcerative lesions on the ventral surface of the same organ. The abdominal cavity showed the presence of a moderate amount of bloody effusion, and the liver and both kidneys appeared slightly enlarged, with a dark-reddish color. At the cut surface, abundant blood was released, indicating moderate to severe, diffuse, renal and hepatic congestion. When the thoracic cavity was opened, the lungs showed bilateral rib marks on the pleural surface and rubbery texture. Additionally, severe emphysema was observed on the dorsal and costal surfaces of the right lung. Finally, a systemic reactive lymph node hyperplasia was reported.

Unfortunately, technical limitations in the preservation and processing of the carcass and tissues impaired the microscopic examination of the available sections, compromising the overall quality of the histopathological interpretation. Nevertheless, some microscopic findings were obtained using specific histochemical staining: in the lungs, the presence of a moderate amount of amorphous, pale eosinophilic material within the alveolar lumen was identified and was indicative of pulmonary edema; moreover, the multifocal, mild to moderate thickening of the alveolar walls due to the presence of mononucleated inflammatory cells was evident, mainly close to small airways, suggesting mild multifocal interstitial pneumonia. Additionally, focal epithelial hyperplasia was noted on sections from the tongue plaque. The histochemical staining did not reveal fat emboli nor mercury pigment in the tested tissues, and the IHC evaluation on the lung tissue did not yield positive results for the detection of CeMV, likely due to the freezing storage of the carcass. 

### 3.2. Biomolecular Analyses

All samples were negative for coronavirus. After the second round of nested RT-PCR, the prescapular lymph node sample turned out to be positive for DMV, and a 158-nucleotide-long sequence (Acc. Numb. PQ186050) was obtained using Sanger sequencing. Of all the DNA extracts, the tongue ulcer and proliferative lesion samples were positive for herpesvirus, and the amplicons were sequenced: a 685-nucleotide-long sequence (Acc. Numb. PQ197058) was obtained from the herpesvirus-positive papillomatous lesion after the first PCR round, and a 415-nucleotide-long herpesvirus sequence (Acc. Numb. PQ197057) was obtained from the tongue ulcer after the second PCR round. 

**Figure 2:**
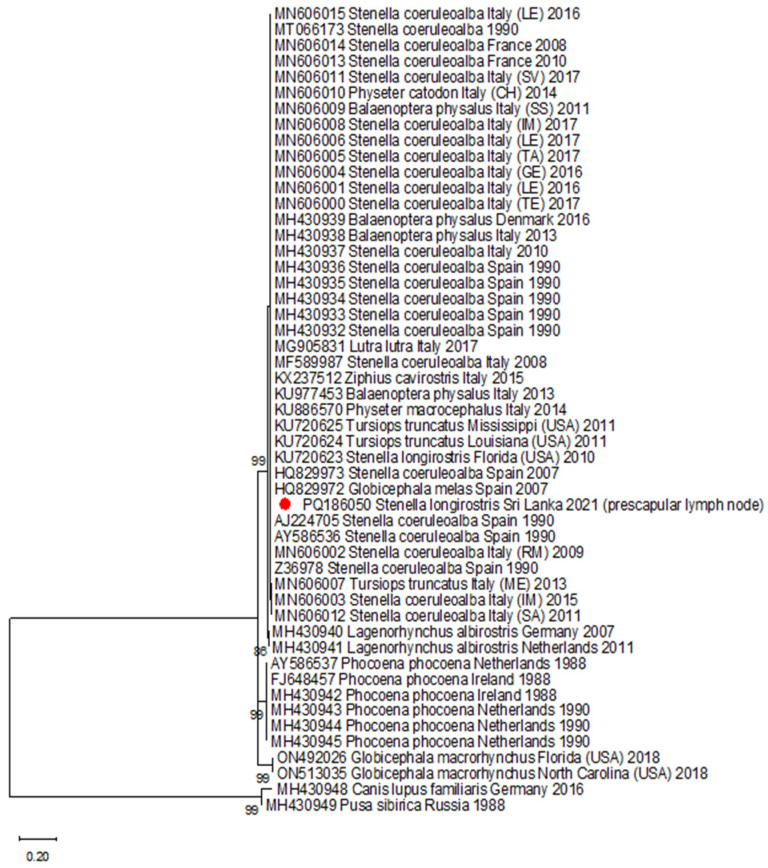
Phylogenetic tree reconstructed using the Maximum Likelihood method and Tamura 3-parameter model with invariable sites. Bootstrap support (≥70%) is shown next to the branches. The analysis involved 51 nucleotide sequences, and a total of 158 positions were considered in the final dataset. The sequence obtained from this study is marked with a red circle.

Thirty-nine DMV, six porpoise morbillivirus (PMV), two pilot whale morbillivirus (PWMV), and two canine morbillivirus (CDV) partial H gene sequences were downloaded from Genbank and merged to the DMV sequence obtained in the present study (Table 1). The database was aligned, trimmed, and used for the reconstruction of a phylogenetic tree (Figure 2).

The sequence belonged to a dolphin morbillivirus strain. Unfortunately, the amplicon was obtained only with the second round of PCR, thus yielding a short sequence and hampering a more in-depth analysis of the strain’s features.

**Figure 3:**
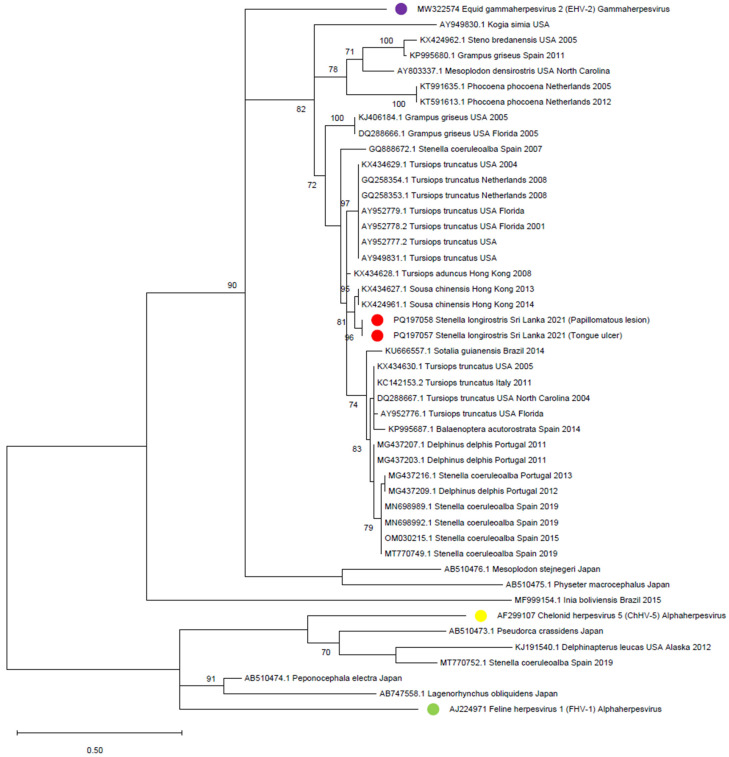
Phylogenetic tree reconstructed using the Maximum Likelihood method and Tamura 3-parameter model with Gamma distribution and invariable sites. Bootstrap support (≥70%) is shown next to the branches. The analysis involved 46 nucleotide sequences, and positions with less than 95% site coverage were eliminated (partial deletion option). Sequences obtained in this study are marked with a red circle, and genus-reference sequences are marked with yellow (Chelonid alphaherpesvirus 5), green (Feline alphaherpesvirus 1), and purple (Equid gammaherpesvirus 2) circles.

Thirty-six cetacean gammaherpesvirus, five cetacean alphaherpesvirus, an Equid gammaherpesvirus 2 (EHV-2), a Chelonid alphaherpesvirus 5 (ChHV-5), and a Feline alphaherpesvirus 1 (FHV-1) partial DNA-directed DNA-polymerase sequences were downloaded from Genbank and merged to the two herpesvirus sequences obtained in the present study (Table 2). The database was aligned, trimmed, and used for the reconstruction of a phylogenetic tree (Figure 3). The sequence obtained from the tongue ulcer was shorter; however, in the overlapping region, it appeared identical to the sequence from the papillomatous lesion. The two strains were characterized as gammaherpesviruses, in a cluster with sequences collected from Indo-Pacific humpback dolphins (*Sousa chinensis)* sampled in Hong Kong.

## 4. Discussion

This study is among the few epidemiological and pathological assessments of stranded marine mammals from Sri Lankan waters, as well as the first detection and characterization study of DMV and herpesvirus from this area. Moreover, to our knowledge, this is one of the few reports of CeMV [40] and herpesvirus detections in the Indian Ocean. The limitations related to logistic conditions, such as the carcass collection, preservation, post-mortem examinations, and tissue sampling, compromised the interpretation of some features and, consequently, the overall systematic health assessment of the case. However, the information on the infection’s presence in the most common dolphin species of Sri Lanka can be considered a baseline for future monitoring projects, implementing the Aquatic One Health model to address marine health issues.

As has often been reported [19,20,41], the coinfection between DMV and herpesvirus was also confirmed in this case, supporting the synergistic action of the two pathogens. DMV and herpesvirus did not seem to have a primary role in the animal’s death, since an interaction with fishing activity appeared to be the most likely cause considering the severity of the gross findings [42,43,44]. 

The two viruses usually cause chronic infections and latency in cetaceans, easing the viral persistence in the individual and spreading within groups of animals. The standardized sampling and testing of stranded animals can enrich knowledge of pathogen presence and circulation in different areas and the assessment of marine mammal health status. Thus, the present work enforces the need for testing not-well-preserved carcasses as well, by resorting to the use of FTA^®^ cards [31]; storage and transport tools based on similar approaches can facilitate disease monitoring in cases where logistics, expertise, or equipment impair post-mortem investigations, increasing global awareness of the epidemiology of emerging pathogens in cetacean conservation, such as morbilliviruses and herpesviruses [8]. Particularly, in this case, without conducting FTA^®^ card sampling and viral detection on the oral lesions, no assumptions of their possible etiology could have been proposed, especially without supportive histological data, which were lacking because of the conditions of the carcass.

Nevertheless, molecular approaches present some limitations, which must be taken into account in the multidisciplinary description of each case and should be flanked by all possible collateral diagnostic assays. Regarding DMV, the short sequence herein obtained hampers the forming of conclusions on the relationships of this strain with others, hindering proper characterization. Moreover, the diagnostic assay was chosen to prioritize sensitivity [36] in reason of the history of the herein-studied individual, its preservation, and the shipment of the samples, but the targeted region (H gene) is different from those sequenced for other strains reported from the Indian Ocean, preventing the comparison and the study of CeMV evolution and dissemination.

Due to the extremely infectious nature of morbilliviruses, the formation and migration of social groups increase the probability of transmission. Because no carrier state has been recognized and infection should confer lifelong immunity [45], large populations of susceptible individuals are required for viral persistence [8]. To further understand disease ecology, metapopulation and multispecies dynamic studies deserve scientific attention and research, especially in less-studied regions. Similarly to European, American, and Australian waters [8], the Indian Ocean appears to host viral circulation and unmonitored outbreaks in cetacean species.

Contrary to DMV, it was possible to place the herein-identified strains into the *Gammaherpesvirus* genus, in a cluster of sequences collected from Indo-Pacific humpback dolphins from Hong Kong (KX424961-KX434627), from which plaque-like lesions on the tongue close to the pharynx, and inside the cranial area of the genital slit on the vaginal mucosa were reported. Interestingly, gammaherpesviruses in cetaceans have often been associated with cutaneous and mucosal lesions [14,15,21,22,23,25,46,47,48,49,50], similar to those on the tongue of the examined individual. However, the limited number of reference sequences could have biased the tree topology and relationship estimations. Unfortunately, the study of these viruses in cetaceans is often hindered by the preservation status of the tissues and thus of the nucleic acids, which are often fragmented, not easily amplified, and even less easily sequenced. Additionally, the low viral titer in samples can affect the length of the sequenced region, especially if the used methods rely on nested PCR to increase sensitivity as in this case, yielding a shorter sequence in the second amplification and limiting the comparability of the sequences. As previously mentioned, different diagnostic methods are commonly employed, with the main aim of sensitivity [51,52,53,54,55], but these methods target different genes [36,56,57] and hamper even more the comparison among all publicly available strains. Considering that herpesvirus infection in cetaceans has been documented since 1988 [58], information about the wide range of diseases caused by these viruses and their ability to cause latency in cetaceans is still scarce. The latter aspect needs dedicated research to understand the virus ecology in cetaceans and the role of human-induced stress in promoting viral reactivation.

These data indicate the presence of DMV and herpesvirus in odontocetes from the Indian Ocean, where mortalities associated to multi-virus infections and mass mortalities have not been reported yet. The collection of preliminary information about viral presence in underinvestigated areas can set the premises for the further monitoring of pathogen epidemiology and marine environmental health, thereby enriching essential knowledge for the management of marine mammal conservation policy in the area. In fact, the recent implementation of an Important Marine Mammal Area (IMMA) in western Sri Lankan waters by the International Union for the Conservation of Nature (IUCN), which also considers *S. longirostris* a relevant species (https://www.marinemammalhabitat.org/factsheets/southwest-east-sri-lanka/ (accessed on 1 September 2024)), represents an important milestone for the monitoring of cetaceans from a comprehensive perspective.

Finally, the constant application of standardized routine post-mortem investigations and frameworks could reveal the anthropic impact on the cetacean population, identifying the evidence of specific harmful fishing gears and suggesting mitigation actions. Furthermore, the establishment of laboratory relationships and investigation networks, supported by tissue banks such as the Mediterranean Marine Mammal Tissue Bank of the BCA Department (https://marinemammals.bca.unipd.it (accessed on 23 May 2022)), can allow routine health surveillance of emerging and re-emerging infectious diseases, which are considered the main natural threats to cetacean conservation worldwide.

## 5. Conclusions

It is difficult to evaluate the contribution of the X-Press Pearl incident to the progress of the reported infections or to the by-catch event. Additional eco-toxicological investigations would be needed to identify the chemical burdens on the animal’s tissues and their potential impact on its health. This case enforces the need for implementing functional stranding networks to investigate marine vertebrate mortalities and conduct passive disease monitoring, in order to account for the anthropogenic impact and the epidemiology of natural infections. A multidisciplinary approach is essential for a systematic assessment of epidemiological data, life history, and environmental parameters, and for providing a complete overview of viral ecology and evolution. The herein-reported case confirms that by-catch and interactions with fishing activities remain major concerns for cetaceans, in addition to the ascertained viral circulation that should be included as another relevant menace to their conservation in Sri Lanka.

## Figures and Tables

**Table 1:** List of accession numbers comprising the sequence database for CeMV phylogenetic analysis.

Acc. Num.	Viral Strain	Host Species	Location	Date
PQ186050	Dolphin morbillivirus	*Stenella longirostris*	Sri Lanka	2021
AY586536	Dolphin morbillivirus	*Stenella coeruleoalba*	Spain	1990
HQ829972	Dolphin morbillivirus	*Globicephala melas*	Spain	2007
HQ829973	Dolphin morbillivirus	*Stenella coeruleoalba*	Spain	2007
KU720623	Dolphin morbillivirus	*Stenella longirostris*	USA (Florida)	2010
KU720624	Dolphin morbillivirus	*Tursiops truncatus*	USA (Louisiana)	2011
KU720625	Dolphin morbillivirus	*Tursiops truncatus*	USA (Mississippi)	2011
KU886570	Dolphin morbillivirus	*Physeter macrocephalus*	Italy	2014
KU977453	Dolphin morbillivirus	*Balaenoptera physalus*	Italy	2013
KX237512	Dolphin morbillivirus	*Ziphius cavirostris*	Italy	2015
MF589987	Dolphin morbillivirus	*Stenella coeruleoalba*	Italy	2008
MG905831	Dolphin morbillivirus	*Lutra lutra*	Italy	2017
MH430932	Dolphin morbillivirus	*Stenella coeruleoalba*	Spain	1990
MH430933	Dolphin morbillivirus	*Stenella coeruleoalba*	Spain	1990
MH430934	Dolphin morbillivirus	*Stenella coeruleoalba*	Spain	1990
MH430935	Dolphin morbillivirus	*Stenella coeruleoalba*	Spain	1990
MH430936	Dolphin morbillivirus	*Stenella coeruleoalba*	Spain	1990
MH430937	Dolphin morbillivirus	*Stenella coeruleoalba*	Italy	2010
MH430938	Dolphin morbillivirus	*Balaenoptera physalus*	Italy	2013
MH430939	Dolphin morbillivirus	*Balaenoptera physalus*	Denmark	2016
MH430940	Dolphin morbillivirus	*Lagenorhynchus albirostris*	Germany	2007
MH430941	Dolphin morbillivirus	*Lagenorhynchus albirostris*	The Netherlands	2011
MN606000	Dolphin morbillivirus	*Stenella coeruleoalba*	Italy (Teramo)	2017
MN606001	Dolphin morbillivirus	*Stenella coeruleoalba*	Italy (Lecce)	2016
MN606002	Dolphin morbillivirus	*Stenella coeruleoalba*	Italy (Rome)	2009
MN606003	Dolphin morbillivirus	*Stenella coeruleoalba*	Italy (Imperia)	2015
MN606004	Dolphin morbillivirus	*Stenella coeruleoalba*	Italy (Genoa)	2016
MN606005	Dolphin morbillivirus	*Stenella coeruleoalba*	Italy (Taranto)	2017
MN606006	Dolphin morbillivirus	*Stenella coeruleoalba*	Italy (Lecce)	2017
MN606007	Dolphin morbillivirus	*Tursiops truncatus*	Italy (Messina)	2013
MN606008	Dolphin morbillivirus	*Stenella coeruleoalba*	Italy (Imperia)	2017
MN606009	Dolphin morbillivirus	*Balaenoptera physalus*	Italy (Sassari)	2011
MN606010	Dolphin morbillivirus	*Physeter catodon*	Italy (Chieti)	2014
MN606011	Dolphin morbillivirus	*Stenella coeruleoalba*	Italy (Savona)	2017
MN606012	Dolphin morbillivirus	*Stenella coeruleoalba*	Italy (Salerno)	2011
MN606013	Dolphin morbillivirus	*Stenella coeruleoalba*	France	2010
MN606014	Dolphin morbillivirus	*Stenella coeruleoalba*	France	2008
MN606015	Dolphin morbillivirus	*Stenella coeruleoalba*	Italy (Lecce)	2016
MT066173	Dolphin morbillivirus	*Stenella coeruleoalba*	-	1990
Z36978	Dolphin morbillivirus	*Stenella coeruleoalba*	Spain	
ON492026	Pilot whale morbillivirus	*Globicephala macrorhynchus*	USA (Florida)	2018
ON513035	Pilot whale morbillivirus	*Globicephala macrorhynchus*	USA (North Carolina)	2018
AY586537	Porpoise morbillivirus	*Phocoena phocoena*	The Netherlands	1988
FJ648457	Porpoise morbillivirus	*Phocoena phocoena*	Ireland	1988
MH430942	Porpoise morbillivirus	*Phocoena phocoena*	Ireland	1988
MH430943	Porpoise morbillivirus	*Phocoena phocoena*	The Netherlands	1990
MH430944	Porpoise morbillivirus	*Phocoena phocoena*	The Netherlands	1990
MH430945	Porpoise morbillivirus	*Phocoena phocoena*	The Netherlands	1990
MH430948	Canine morbillivirus	*Canis lupus familiaris*	Germany	2016
MH430949	Canine morbillivirus	*Pusa sibirica*	Russia	1988

**Table 2:** List of accession numbers comprising the sequence database for herpesvirus phylogenetic analysis.

Acc. Numb.	Genus/Species	Host Species	Location	Date
PQ197057	*Gammaherpesvirus*	*Stenella longirostris*	Sri Lanka	2021
PQ197058	*Gammaherpesvirus*	*Stenella longirostris*	Sri Lanka	2021
AB510475.1	*Gammaherpesvirus*	*Physeter macrocephalus*	Japan	
AB510476.1	*Gammaherpesvirus*	*Mesoplodon stejnegeri*	Japan	
AY803337.1	*Gammaherpesvirus*	*Mesoplodon densirostris*	USA (North Carolina)	
AY949830.1	*Gammaherpesvirus*	*Kogia simia*	USA	
AY949831.1	*Gammaherpesvirus*	*Tursiops truncatus*	USA	
AY952776.1	*Gammaherpesvirus*	*Tursiops truncatus*	USA (Florida)	
AY952777.2	*Gammaherpesvirus*	*Tursiops truncatus*	USA	
AY952778.2	*Gammaherpesvirus*	*Tursiops truncatus*	USA (Florida)	2001
AY952779.1	*Gammaherpesvirus*	*Tursiops truncatus*	USA (Florida)	
DQ288666.1	*Gammaherpesvirus*	*Grampus griseus*	USA (Florida)	2005
DQ288667.1	*Gammaherpesvirus*	*Tursiops truncatus*	USA (North Carolina)	2004
GQ258353.1	*Gammaherpesvirus*	*Tursiops truncatus*	The Netherlands	2008
GQ258354.1	*Gammaherpesvirus*	*Tursiops truncatus*	The Netherlands	2008
GQ888672.1	*Gammaherpesvirus*	*Stenella coeruleoalba*	Spain	2007
KC142153.2	*Gammaherpesvirus*	*Tursiops truncatus*	Italy	2011
KJ406184.1	*Gammaherpesvirus*	*Grampus griseus*	USA	2005
KP995680.1	*Gammaherpesvirus*	*Grampus griseus*	Spain	2011
KP995687.1	*Gammaherpesvirus*	*Balaenoptera acutorostrata*	Spain	2014
KT591613.1	*Gammaherpesvirus*	*Phocoena phocoena*	The Netherlands	2012
KT991635.1	*Gammaherpesvirus*	*Phocoena phocoena*	The Netherlands	2005
KU666557.1	*Gammaherpesvirus*	*Sotalia guianensis*	Brazil	2014
KX424961.1	*Gammaherpesvirus*	*Sousa chinensis*	Hong Kong	2014
KX424962.1	*Gammaherpesvirus*	*Steno bredanensis*	USA	2005
KX434627.1	*Gammaherpesvirus*	*Sousa chinensis*	Hong Kong	2013
KX434628.1	*Gammaherpesvirus*	*Tursiops aduncus*	Hong Kong	2008
KX434629.1	*Gammaherpesvirus*	*Tursiops truncatus*	USA	2004
KX434630.1	*Gammaherpesvirus*	*Tursiops truncatus*	USA	2005
MF999154.1	*Gammaherpesvirus*	*Inia boliviensis*	Brazil	2015
MG437203.1	*Gammaherpesvirus*	*Delphinus delphis*	Portugal	2011
MG437207.1	*Gammaherpesvirus*	*Delphinus delphis*	Portugal	2011
MG437209.1	*Gammaherpesvirus*	*Delphinus delphis*	Portugal	2012
MG437216.1	*Gammaherpesvirus*	*Stenella coeruleoalba*	Portugal	2013
MN698989.1	*Gammaherpesvirus*	*Stenella coeruleoalba*	Spain	2019
MN698992.1	*Gammaherpesvirus*	*Stenella coeruleoalba*	Spain	2019
MT770749.1	*Gammaherpesvirus*	*Stenella coeruleoalba*	Spain	2019
OM030215.1	*Gammaherpesvirus*	*Stenella coeruleoalba*	Spain	2015
MW322574	Equid gammaherpesvirus 2 (EHV-2)			
AF299107	Chelonid herpesvirus 5 (ChHV-5)			
AJ224971	Feline herpesvirus 1 (FHV-1)			
AB510473.1	*Alphaherpesvirus*	*Pseudorca crassidens*	Japan	
AB510474.1	*Alphaherpesvirus*	*Peponocephala electra*	Japan	
AB747558.1	*Alphaherpesvirus*	*Lagenorhynchus obliquidens*	Japan	
KJ191540.1	*Alphaherpesvirus*	*Delphinapterus leucas*	USA (Alaska)	2012
MT770752.1	*Alphaherpesvirus*	*Stenella coeruleoalba*	Spain	2019

## Data Availability

Data is contained within the article and in a publicly accessible repository.
